# Case report: Interstitial implantation radiotherapy combined with immunotherapy and GM-CSF in oligometastatic platinum-resistant ovarian cancer

**DOI:** 10.3389/fimmu.2023.1329951

**Published:** 2024-01-03

**Authors:** Yi Qin, Shangke Huang, Junli Tang, Yu Fan, Xiangyu Deng, Ping Guan, Zhenhua Zhang, Qinglian Wen, Dan Li

**Affiliations:** ^1^ Department of Oncology, The Affiliated Hospital of Southwest Medical University, Southwest Medical University, Luzhou, Sichuan, China; ^2^ Department of Oncology, The First People’s Hospital of Suining, Suining, Sichuan, China; ^3^ Department of Radiation Oncology, Cancer Center, West China Hospital, Sichuan University, Chengdu, Sichuan, China

**Keywords:** platinum-resistant ovarian cancer, interstitial implantation radiotherapy, immunotherapy, GM-CSF, PRaG therapy, case report

## Abstract

**Background:**

Treatment for platinum-resistant ovarian cancer is challenging. Currently, platinum-resistant ovarian cancer is typically treated with non-platinum single-agent chemotherapy ± bevacizumab, but the prognosis is often extremely poor. In the treatment of platinum-resistant ovarian cancer patients, reports of triple therapy with interstitial implantation radiotherapy combined with immunotherapy and granulocyte-macrophage colony-stimulating factor (GM-CSF) (PRaG for short) are relatively rare.

**Case description:**

Here, we report a patient with oligometastatic platinum-resistant ovarian cancer. The patient achieved partial response (PR) of the lesion and sustained benefit for more than six months after receiving interstitial implantation radiotherapy combined with immunotherapy along with GM-CSF.

**Conclusion:**

This triple therapy may provide additional options for these patients.

## Introduction

Ovarian cancer is the second leading cause of death among women from gynecologic malignancies worldwide (1). The majority of ovarian cancer patients are at an advanced stage once confirmed, and the standard of care for advanced ovarian cancer (International Federation of Gynecology and Obstetrics FIGO stage III-IV) is tumor cytoreduction and chemotherapy based on platinum and paclitaxel drugs (2). Platinum-resistant ovarian cancer is a heterogeneous illness with a very bad prognosis and limited survival which commonly advances within 6 months of completing platinum-based therapy. It usually has a survival period of less than 18 months (3). At first recurrence, platinum resistance occurs in about 20% of patients and almost all recurrent patients eventually move toward platinum resistance (4). Currently, platinum-resistant ovarian cancer is typically treated with non-platinum single-agent chemotherapy ± bevacizumab. Non-platinum single-agent chemotherapy has an overall response rate of just 10–15%, a progression-free survival (PFS) of only 4 months, and an overall survival (OS) of roughly 12 months for patients with platinum-resistant ovarian cancer (5). AURELIA clinical trial showed a significant increase in response rates in platinum-resistant patients when combined with bevacizumab, but median survival did not exceed 16 months (6).

Ovarian cancer is immunogenic with immunotherapy promising a role in platinum-resistant ovarian cancer (7, 8). However, programmed cell death protein 1 receptor (PD-1)/PD-1 ligand (PD-L1) antibodies monotherapy has a low response rate in platinum-resistant ovarian cancer, typically no more than 8% (3).Therefore, it is crucial to explore novel approaches to sensitization immunotherapy. Multimodal therapeutic strategies are being investigated to enhance anti-PD1/PD-L1 response rates by the combination of chemotherapy, antiangiogenic agents, radiotherapy, or other immune checkpoint inhibitors (3). Among them, combining stereotactic body radiation therapy (SBRT), hypofractionated radiation therapy (HFRT) or brachytherapy (BT) may be a prospective therapeutic strategy (3, 9, 10).

Here, we present a case of an oligometastatic platinum-resistant ovarian cancer patient. The patient received triple therapy with interstitial implantation radiotherapy combined with immunotherapy and GM-CSF (PRaG for short). At the end of treatment, the patient achieved a PR and sustained benefit for more than 6 months.

## Case description

**Figure 1:**
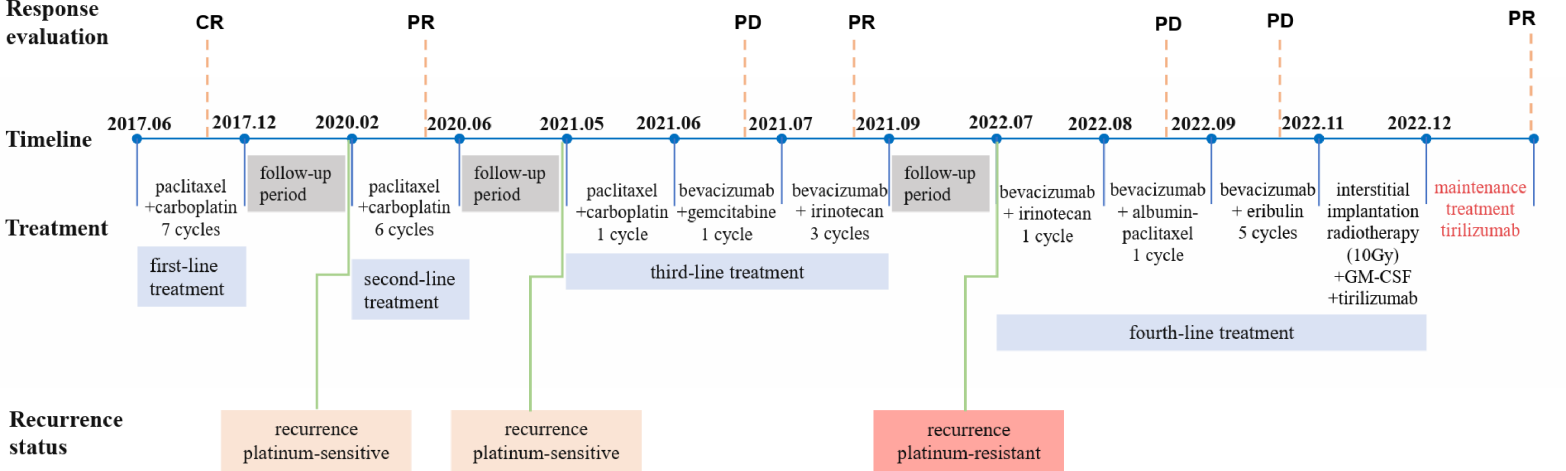
Timeline of different treatments and disease status.

We show the treatment timeline for the patient in **Figure 1**. In June 2017 (Sichuan, China), a 66-year-old woman was admitted to our hospital with abdominal distension for more than 6 months. The patient had a total of four pregnancies, three abortions and one normal delivery. Ascites cytology result showed malignant cells (poorly differentiated, considered adenocarcinoma). An abdominal computed tomography (CT) scan revealed bilateral adnexal masses and multiple retroperitoneal lymph nodes. The cancer antigen 125 (CA 125) blood test was 698.20 U/ml. Based on the above clinical results, the patient was diagnosed with ovarian adenocarcinoma (FIGO 2017 Stage IIIC). The patient refused surgery for personal reasons and underwent 6 cycles of chemotherapy (paclitaxel 175 mg/m^2^ + carboplatin AUC=5, ivgtt, q21d). After completion of 6 cycles of chemotherapy, abdominal CT confirmed a complete response (CR). The physician again recommended surgical resection, which the patient declined. Considering the patient’s actual condition, the oncologist implemented the 7th cycle of chemotherapy (paclitaxel 175 mg/m2 + carboplatin AUC=5, ivgtt, q21d). Upon completion of the treatment, follow-up abdominal CT and tumor markers (CA125 and human epitope protein 4 (HE4)) did not show any signs of recurrence for more than 2 years.

In February 2020, the patient presented for vaginal bleeding. A pelvic magnetic resonance imaging (MRI) revealed a cystic solid mass shadow in the pelvis (size 10.3×6.3×8.2 cm). CA125 was greater than 1000 U/ml and HE4 was 114.90 pmol/L. The oncologist considered the patient to be a platinum-sensitive recurrence. The patient still refused surgery and underwent a second course of 6 cycles of systemic chemotherapy (paclitaxel 175 mg/m^2^ + carboplatin AUC=5, ivgtt, q21d). CT confirms that localized lesions achieve PR and CA125 consistently decreases to the normal range (23.30 - >1000 U/ml). After the completion of chemotherapy, the oncologist advised the patient to perform surgical resection or maintenance therapy, but the patient refused all therapeutic recommendations.

Afterward, the patient progressed again in less than a year, still presenting as a localized adnexal mass and same location as the first recurrence. MRI (May 2021) showed a cystic solid mass shadow in the pelvis (size 12.2×7.9×10.0 cm) and CA125 was 560.05 U/ml and HE4 was 186.10 pmol/L. The oncologist considered the patient a platinum-sensitive recurrence again. However, after 1 cycle of chemotherapy (paclitaxel 175 mg/m^2^ + carboplatin AUC=5, ivgtt, q21d), the patient’s CA125 remained elevated (855.34 U/ml). Positron emission tomography-computed tomography (PET-CT) demonstrated there is a huge mass in the pelvic cavity with increased glucose metabolism, compared with the pelvic MRI in May 2021, the volume of the pelvic lesion has slightly increased. Considered platinum-resistant, it was replaced with bevacizumab combined with gemcitabine (bevacizumab 7.5 mg/kg + gemcitabine 1.0 g/m^2^, d1, d8 ivgtt, q21d) in June 2021. As the tumor marker serum CA125 continued to rise, the oncologist implemented 3 cycles of targeted drug combination chemotherapy (bevacizumab 7.5mg/kg d1 + irinotecan 80mg/kg d1, d8, d15 q21d). The last systemic treatment was in September 2021. MRI in October 2021 suggested a significant reduction in the shadow of the cystic solid mass in the pelvis, and efficacy was evaluated as PR of the localized lesion.

**Figure 2:**
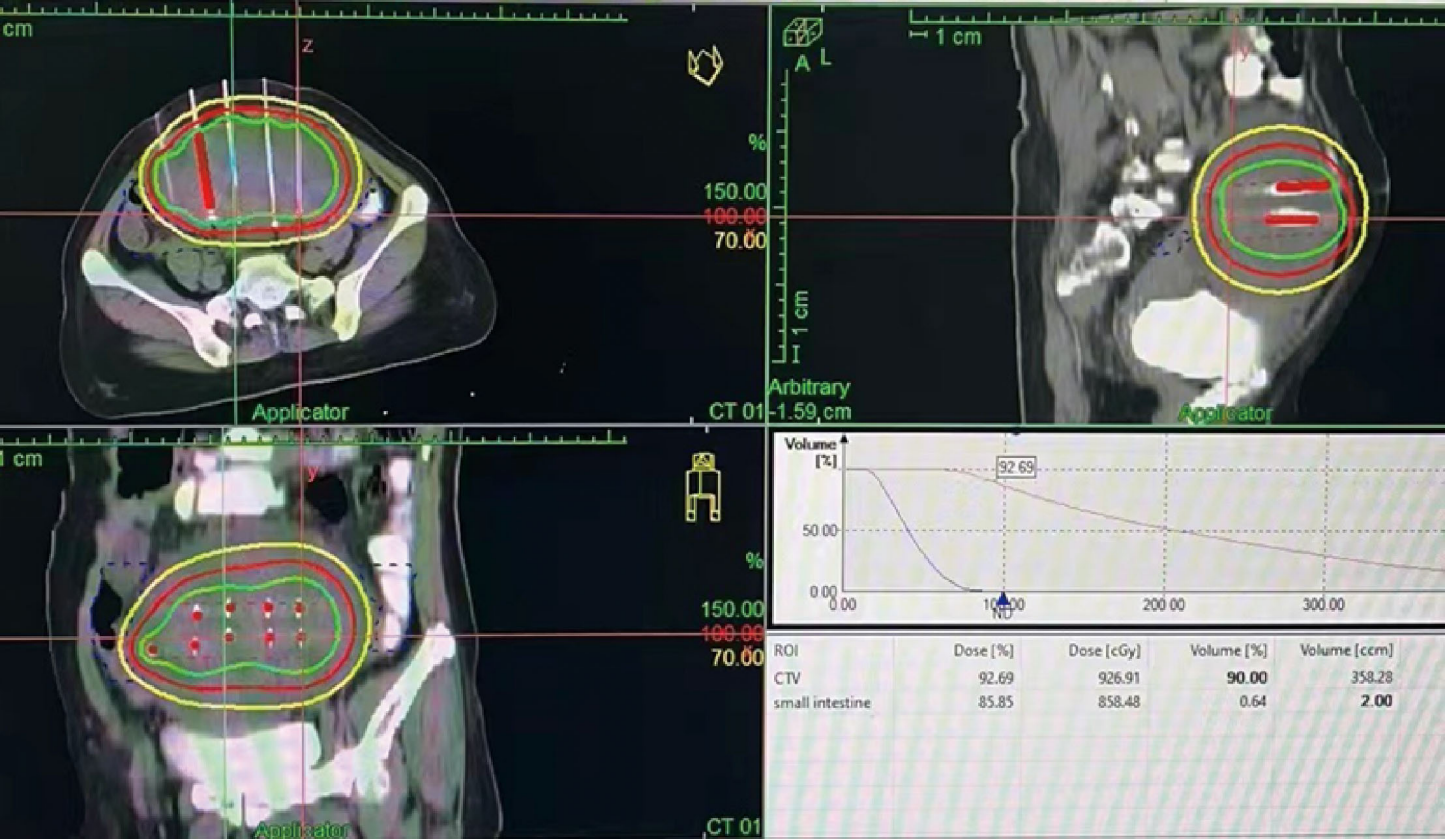
Interstitial implantation radiotherapy: Three-dimensional conformal dose assessment.

**Figure 3:**
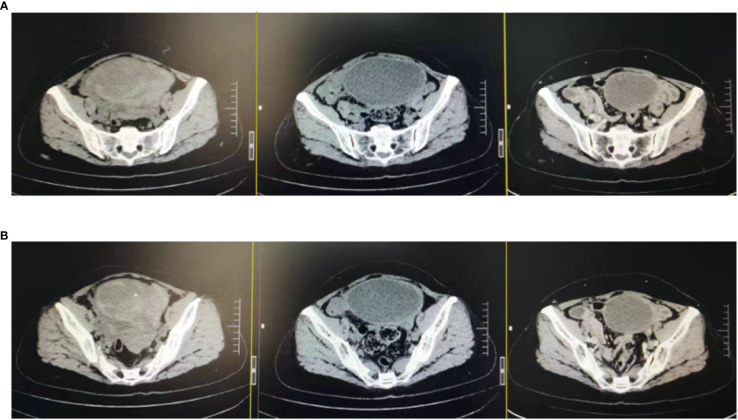
Changes in lesions: After the triple therapy, the CT showed a significantly smaller pelvic mass. **(A)** Pelvic mass before the triple therapy. **(B)** Pelvic mass after the triple therapy.

**Figure 4:**
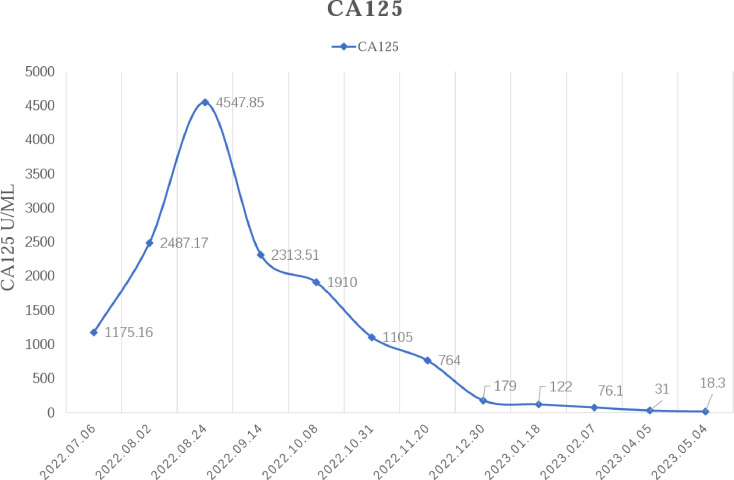
Changes in serum CA125 levels before and after the triple therapy.

In February 2022, the patient experienced abdominal distension again along with a large amount of ascites. In July 2022, the patient was readmitted to our hospital with a worsening condition. Abdominal CT suggested a cystic solid mass in the pelvis (size 12.5×10.6 cm) and CA125 was 1175.16 U/ml and HE4 was 133.80 pmol/L. Tumor recurrence was considered. From July 2022 to November 2022, the patient received 7 cycles of systemic therapy with a targeted agent in combination with a chemotherapeutic agent. On the clinician’s recommendation, the patient received 1 cycle of bevacizumab in combination with irinotecan (bevacizumab 7.5 mg/kg + irinotecan 60 mg/kg, ivgtt, q21d), 1 cycle of bevacizumab in combination with albumin-paclitaxel (bevacizumab 7.5 mg/kg + albumin-paclitaxel q21d), and 5 cycles of bevacizumab in combination with eribulin (bevacizumab 7.5mg/kg + eribulin 2mg d1, d8, q21d). During the treatment, the pelvic mass of the patient was still increasing, and the general condition was getting worse. The patient refused to undergo palliative surgery to relieve symptoms and to be enrolled in clinical studies. Considering that the patient has experienced multiple relapses with the same pelvic lesion and the lesion is isolated, local radiotherapy combined with immunotherapy was chosen. Due to financial reasons, the patient refused immune-related genetic tests, including microsatellite instability (MSI) status, programmed cell death-ligand 1 (PD-L1), and tumor mutation burden (TMB). However, considering the MSI-H/dMMR incidence of up to 30% (11), our patient strongly expressed her willingness to do immunotherapy and chose the relatively affordable and cheap the PD-1 inhibitor tirilizumab produced in China. We informed our patient of the treatment purpose and risks, and signed an informed consent form. The patient then received triple therapy from November 30, 2022. The radiation oncologist implemented interstitial implantation radiotherapy at a prescribed dose of 10 Gy, combined with a subcutaneous injection of GM-CSF (200 µg) for one week. The tumor got an actual dose of 926.91 cGy. On December 2, 2022, the patient began immunotherapy with the PD-1 inhibitor tirilizumab (300 mg, ivgtt). **Figure 2** shows the three-dimensional conformal dose assessment for interstitial implantation radiation therapy. After radiotherapy, the patient developed mild localized erythema. The patient now has no skin ulcers, no bilateral lower extremity edema or other complications, and only mild localized skin pigmentation. The patient’s efficacy evaluation showed a PR. After that, single-agent maintenance therapy with the PD-1 inhibitor tirilizumab was administered every three weeks. During immune maintenance therapy, the patient was temporarily free of treatment-related adverse events (TRAEs), like hemopoietic, thyroid, lung, and heart dysfunction. As of the follow-up in June 2023, abdominal CT suggested a smaller pelvic mass than before (**Figure 3**) and CA125 was persistently decreasing (most recent CA125 was 18.30 U/ml) (**Figure 4**). The patient’s lesion achieved a PR and continues to benefit for more than six months.

## Discussion

Currently, the overall outcome of PD-1/PD-L1 antibody therapy in recurrent ovarian cancer is not good. The use of a single immune checkpoint inhibitor has shown relatively low response rates, usually in the range of 10-15% (12). The response rates to single-agent immunotherapy in platinum-resistant ovarian cancer are even lower (3). Several clinical trials on immune checkpoint inhibitors to platinum-resistant ovarian cancer are ongoing. The JAVELIN phase I clinical study revealed an objective remission rate (ORR) of 9.6% for 125 patients after monotherapy with Avelumab (13). In the phase II clinical study of KEYNOTE-100, the investigators designed two cohorts that had ORRs of 7.4% and 9.9% after treatment with pembrolizumab (14). Several data have shown that combination therapy with immune checkpoint inhibitors shows some advantages over monotherapy. The clinical study of KEYNOTE-162 showed that pembrolizumab and the PARP inhibitor niraparib together had an ORR of 18% for the treatment of platinum-resistant recurrent ovarian cancer (15). Phase II clinical study NCT02853318 evaluated pembrolizumab in combination with bevacizumab and oral cyclophosphamide for recurrent ovarian cancer with an ORR of 47.5% (16). However, the above clinical trials on combination therapy with immune checkpoint inhibitors are small sample studies and their results need to be further validated.

Radiation therapy’s clinical success has been linked to the ability of ionizing radiation to cause DNA damage, which can instantly kill tumor cells. However, ionizing radiation can also produce a non-DNA-targeted radiation effect (17). Radiation therapy can initiate the immune system through T-cell mediation. Irradiation-induced immunomodulation can affect both irradiated tumor cells and have an effect on the tumor immune microenvironment (18, 19). Radiation-induced immune stimulation generates a series of molecular reactions through both local and systemic immune mediators, resulting in the creation of a pro-inflammatory environment (20). Irradiation can increase the expression of MHC-I and MHC-II molecules, adhesion molecules, CD80, stress ligands and death receptors on the surface of tumor cells, simultaneously releasing immune-activating danger signals, chemokines, inflammatory cytokines, and possibly even inducing new tumor antigens, which triggers a systemic response (21–23). In this process, mature dendritic cells (DCs) are activated and stimulate the innate immune system, indirectly generating an adaptive immune response (21). It means that tumor cells may be transformed into *in situ* vaccines under irradiation-induced immune stimulation, exerting local tumor control and possibly triggering the so-called “abscopal effect” at distant tumor sites (17, 24). And, immune checkpoint inhibitors act synergistically with radiation therapy to boost local tumor control and systemic response (25). Irradiated tumor cells undergo a specific form of cell death (so-called immune cell death). This cell death exposes tumor cell-associated antigens, allowing for synergy with immunotherapy (26, 27). Thus, radiation therapy combined with immune therapy has been more and more recognized as a possible treatment strategy.

Pelvic irradiation is not included in the National Comprehensive Cancer Network (NCCN) guideline for platinum-resistant ovarian cancer patients (28). However, data have shown that for recurrent ovarian cancer patients, the median survival after HFRT is 17 months, the 1-year survival rate is 66.7% and the 1-year local progression-free survival rate is 45.8% (29). HFRT may be an alternative therapy. In the case of oligometastases, SBRT is a novel high-dose radiation beam treatment. In a study of oligometastatic platinum-resistant ovarian cancer, 156 lesions treated with SBRT were evaluated radiologically. 91 (58%) lesions showed a complete radiologic response, 26 (17%) lesions showed a partial response, 24 (15%) had stable disease, and 11 (7%) showed disease progression (30). Moreover, SBRT has been shown in several clinical trials to have a local control rate of 90-100% in oligometastatic platinum-resistant patients (31, 32). These studies proved the radiosensitivity of platinum-resistant ovarian cancer. Since our patient had a large pelvic lesion, it was difficult to maneuver the SBRT. We chose interstitial implantation radiotherapy for the patient, which used the technique of large fractionation radiotherapy, and only a dose of 10 Gy was given to activate the immune T-cells and synergize with the subsequent immunotherapy. Few reports on interstitial implantation brachytherapy for recurrent ovarian cancer have been reported. In a retrospective study, 47 recurrent ovarian cancer patients were treated with brachytherapy, and the local control rates at 3, 6, 12, 24, and 36 months were 93.3%, 77.7%, 58.9%, 38.7%, and 19.3%, respectively, and the mean OS of 14.6 months (33). Although there are few reports on the local control rate of interstitial implantation radiotherapy for platinum-resistant ovarian cancer, we believe that this treatment is feasible.

GM-CSF is a cytokine which drives the production of myeloid cell subsets including neutrophils, monocytes, macrophages, and dendritic cells (34). Preclinical studies have demonstrated that GM-CSF in combination with immune checkpoint inhibitors enhances innate immune cell activity and indirectly recruits T-cells by promoting antigen cross-presentation, thereby enhancing the immune response (35, 36). PD-1 inhibitors combined with radiotherapy or/and GM-CSF can have a synergistic effect (37–42). Triple-combination therapy of these treatments was called PRaG for short. A clinical trial demonstrated that concurrent radiotherapy with pembrolizumab dramatically enhanced response and prognosis in patients with non-small cell lung carcinoma (median OS: 19.2 months vs. 8.7 months, PFS: 9.0 months vs. 4.4 months) (41). In unresectable advanced melanoma patients, ibritumomab combined with GM-CSF resulted in longer survival and fewer toxic side effects than ibritumomab alone (43). In a phase II clinical trial for refractory metastatic solid tumors, 54 patients were treated with PRaG therapy, resulting in an ORR of 16.7%, a disease control rate of 46.3%, and a median PFS of 4.0 months (42). Consistent with the results of these clinical studies, we have achieved favorable outcomes using interstitial implantation radiotherapy in combination with a PD-1 inhibitor and GM-CSF. By June 2023, the patient’s efficacy evaluation was a PR and the general condition has significantly improved and the patient is continuing to benefit.

The most regrettable aspect of this study is that we did not conduct a coarse needle biopsy to obtain tissue pathological diagnosis before undergoing local radiotherapy. We have reason to believe that our patient should be a special case of ovarian cancer, with multiple relapses occurring in the same location. The patient achieved good results in a single fractionated radiotherapy combined with immunotherapy, but the gene expression related to immunotherapy is not very clear. Although our treatment was similar to PRaG therapy, PRaG therapy is usually repeated several times to adequately induce the immune response, which was only done once in this study. Despite these disadvantages, we were pleasantly surprised to find that the triple therapy of single high-dose interstitial implantation radiotherapy for large isolated pelvic masses in combination with immunotherapy and GM-CSF achieved a longer disease progression-free time, which the mechanism of this deserves to be further explored.

## Conclusion

In conclusion, oligometastatic platinum-resistant ovarian cancer patients who fail to receive bevacizumab in combination with non-platinum monotherapy tend to have an extremely poor prognosis, and subsequent treatment becomes tricky. In our case, the patient was treated with a triple combination of interstitial implantation radiotherapy, PD-1 inhibitor immunotherapy, and GM-CSF, showing a sustained clinical response. Moreover, the patient had only minor toxic side effects. This might offer a novel treatment option for similar patients.

## Data availability statement

The original contributions presented in the study are included in the article/supplementary material. Further inquiries can be directed to the corresponding authors.

## Ethics statement

Written informed consent was obtained from the individual(s) for the publication of any potentially identifiable images or data included in this article.

## Author contributions

YQ: Visualization, Writing – original draft. SH: Writing – original draft. JT: Writing – original draft. YF: Writing – review & editing. XD: Writing – review & editing. PG: Writing – review & editing. ZZ: Supervision, Writing – review & editing. QW: Supervision, Writing – review & editing. DL: Conceptualization, Supervision, Writing – review & editing.
